# *meso*-[5,5,7,12,12,14-Hexamethyl-1,4,8,11-tetra­azacyclo­tetra­deca-1(14),7-diene]nickel(II) dibromide dihydrate

**DOI:** 10.1107/S1600536810016831

**Published:** 2010-05-19

**Authors:** Feifei Shi, Xiaodan Chen, Rong Rong, Qianqian Bao

**Affiliations:** a Ordered Matter Science Research Center, College of Chemistry and Chemical Engineering, Southeast University, Nanjing 210096, People’s Republic of China; b Department of Chemistry, Key Laboratory of Medicinal Chemistry for Natural Resource, Ministry of Education, Yunnan University, Kunming 650091, People’s Republic of China

## Abstract

The asymmetric unit of the title complex, [Ni(C_16_H_32_N_4_)]Br_2_·2H_2_O, contains two [Ni(C_16_H_32_N_4_)]^2+^ cations, four Br^−^ anions and four uncoordinated H_2_O mol­ecules. The Ni atoms are in a slightly distorted square-planar coordination by the four macrocyclic N atoms, which are almost coplanar [N—N—N—N torsion angles of 2.97 (6) and 3.12 (7)°]. In the crystal, a network of N—H⋯Br, O—H⋯Br and N—H⋯O hydrogen bonds leads to the formation of a chain structure.

## Related literature

The nickel (II) tetra­azamacrocyclic complex cation, [Ni(C_16_H_32_N_4_)]^2+^, has both meso and enanti­omeric forms, see: Warner *et al.* (1968 ▶). For the structures of related macrocyclic complexes, see: Whimp *et al.* (1970 ▶). For Ni—N(amine) and Ni—N(imine) bond distances, see: Szalda *et al.* (1991 ▶).
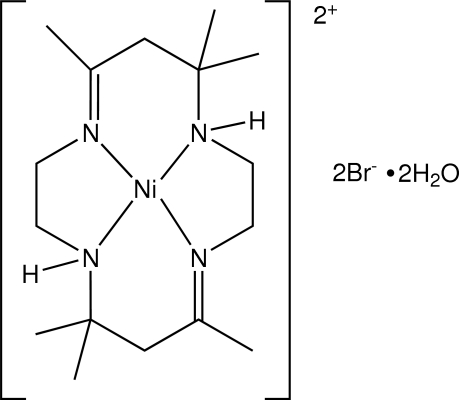


## Experimental

### 

#### Crystal data

[Ni(C_16_H_32_N_4_)]Br_2_·2H_2_O
*M* *_r_* = 535.02
Monoclinic, 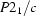

*a* = 17.8712 (15) Å
*b* = 15.5028 (12) Å
*c* = 17.2324 (17) Å
β = 112.077 (1)°
*V* = 4424.2 (7) Å^3^
*Z* = 8
Mo *K*α radiation
μ = 4.51 mm^−1^
*T* = 293 K
0.27 × 0.20 × 0.20 mm

#### Data collection

Rigaku SCXmini diffractometer
Absorption correction: multi-scan (*CrystalClear*; Rigaku, 2005 ▶) *T*_min_ = 0.831, *T*_max_ = 0.862
22003 measured reflections
7779 independent reflections
4053 reflections with *I* > 2σ(*I*)
*R*_int_ = 0.058

#### Refinement

*R*[*F* ^2^ > 2σ(*F* ^2^)] = 0.038
*wR*(*F* ^2^) = 0.059
*S* = 0.96
7779 reflections
463 parameters
H-atom parameters constrained
Δρ_max_ = 0.64 e Å^−3^
Δρ_min_ = −0.66 e Å^−3^

### 

Data collection: *CrystalClear* (Rigaku, 2005 ▶); cell refinement: *CrystalClear*; data reduction: *CrystalClear*; program(s) used to solve structure: *SHELXS97* (Sheldrick, 2008 ▶); program(s) used to refine structure: *SHELXL97* (Sheldrick, 2008 ▶); molecular graphics: *SHELXTL* (Sheldrick, 2008 ▶); software used to prepare material for publication: *SHELXTL*.

## Supplementary Material

Crystal structure: contains datablocks I, global. DOI: 10.1107/S1600536810016831/vm2024sup1.cif

Structure factors: contains datablocks I. DOI: 10.1107/S1600536810016831/vm2024Isup2.hkl

Additional supplementary materials:  crystallographic information; 3D view; checkCIF report

## Figures and Tables

**Table:** 

Ni2—N6	1.976 (4)
Ni2—N8	1.983 (3)
Ni2—N7	2.003 (3)
Ni2—N5	2.029 (3)
Ni1—N2	1.985 (4)
Ni1—N4	1.993 (4)
Ni1—N1	2.007 (3)
Ni1—N3	2.020 (3)

**Table:** 

N6—Ni2—N8	172.20 (16)
N6—Ni2—N7	84.38 (14)
N8—Ni2—N7	94.04 (14)
N6—Ni2—N5	94.20 (14)
N8—Ni2—N5	85.74 (14)
N7—Ni2—N5	167.87 (17)
N2—Ni1—N4	174.32 (16)
N2—Ni1—N1	94.09 (14)
N4—Ni1—N1	85.03 (14)
N2—Ni1—N3	85.80 (14)
N4—Ni1—N3	94.09 (14)
N1—Ni1—N3	170.06 (17)

**Table 2:** Hydrogen-bond geometry (Å, °)

*D*—H⋯*A*	*D*—H	H⋯*A*	*D*⋯*A*	*D*—H⋯*A*
N1—H1⋯Br3	0.91	2.55	3.444 (4)	167
O2—H2*F*⋯Br2	0.85	2.60	3.437 (3)	171
O3—H3*D*⋯Br3	0.85	2.63	3.473 (3)	171
N5—H5⋯O4^i^	0.91	2.41	3.269 (5)	158
O1—H1*C*⋯Br1^i^	0.85	2.55	3.390 (3)	170
N7—H7⋯Br2^ii^	0.91	2.54	3.436 (4)	169
N3—H3⋯O1^ii^	0.91	2.45	3.328 (5)	161
O2—H2*E*⋯Br3^iii^	0.85	2.50	3.339 (4)	171
O1—H1*D*⋯Br4^iii^	0.85	2.44	3.280 (3)	169
O3—H3*C*⋯Br2^ii^	0.85	2.48	3.316 (3)	170
O4—H4*G*⋯Br4^ii^	0.85	2.49	3.335 (4)	171
O4—H4*F*⋯Br1^iv^	0.85	2.39	3.228 (3)	171

Symmetry codes: (i) 
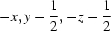
; (ii) 
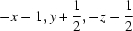
; (iii) 
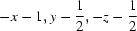
; (iv) 
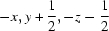
.

**Fig. 1.**
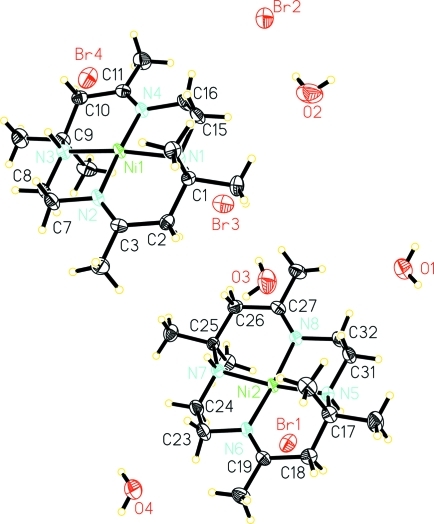
The asymmetric structure of the title molecule, with the atom-numbering scheme. Displacement ellipsoids are drawn at the 30% probability level.

**Fig. 2.**
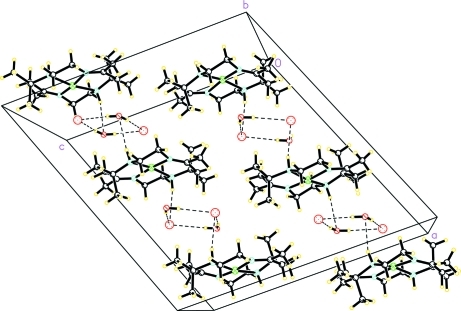
Crystal packing of the compound (I) (viewed along the b axis). Hydrogen bonds are shown as dashed lines.

## supplementary crystallographic information

### Comment

The structures of several related macrocyclic complexes have been reported (Whimp *et al.*, 1970). The nickel (II) tetraazamacrocyclic complex cation, [Ni(C_16_H_32_N_4_)]^2+^, has both meso and enantiomeric forms (Warner *et al.*, 1968) and can combine with different anions to form many kinds of structures.

We herein report the crystal structure of a new compound synthesized by the reaction of Ni(NO_3_)_2_.6H_2_O and the complex C_18_H_32_N_4_.2HBr.2H_2_O. Two similar macrocycles are included in the asymmetric unit, and the crystal structure is stabilized by intermolecular hydrogen bonds. As the two macrocycles are in similar coordination with nickel(II), only one of them will be described. As shown in Fig.1, the Ni^II^ atom is coordinated by four N atoms from the tetraazamacrocycle in a square-planar geometry, the average Ni—*N*(amine) bond distance of 2.0132 (4) Å and Ni—*N*(imine) bond distance of 1.9899 (4)Å are similar to those previously reported (Szalda *et al.*,1991). The dihedral "fold" angle between the planes formed by N1, N2, N3 and N1, N3, N4 is 4.343 (1)°. The macrocycle is in the N-meso configuration with the amine hydrogens of N1 and N4 on opposite sides of the macrocycle plane. The combination of [Ni(C_16_H_32_N_4_)]^2+^with two Br^-^ anions and two isolated H_2_O established the title compound.

### Experimental

All chemicals were of reagent grade and were used as received without further purification. The precursor complex C_18_H_32_N_4_.2HBr.2H_2_O was prepared previously. To a 10 ml methanol solution of Ni(NO_3_)_2_.6H_2_O (0.2 mmol, 0.344 g), a 5 ml methanol solution of C_18_H_32_N_4_.2HBr.2H_2_O (0.2 mmol, 0.0957 g) was added dropwise with stirring. The resulting solution was stirred continuously for about 30 min. Brown crystals suitable for X-ray analysis were obtained by slow evaporation at room temperature over several days.

### Refinement

(type here to add refinement details)

### Crystal data

**Table:**

[Ni(C_16_H_32_N_4_)]Br_2_·2H_2_O	*F*(000) = 2192
*M**_r_* = 535.02	*D*_x_ = 1.606 Mg m^−^^3^
Monoclinic, *P*2_1_/*c*	Mo *K*α radiation, λ = 0.71073 Å
Hall symbol: -P 2ybc	Cell parameters from 3476 reflections
*a* = 17.8712 (15) Å	θ = 2.3–27.5°
*b* = 15.5028 (12) Å	µ = 4.51 mm^−^^1^
*c* = 17.2324 (17) Å	*T* = 293 K
β = 112.077 (1)°	Prism, brown
*V* = 4424.2 (7) Å^3^	0.27 × 0.20 × 0.20 mm
*Z* = 8	

### Data collection

**Table:**

Rigaku SCXmini diffractometer	7779 independent reflections
Radiation source: fine-focus sealed tube	4053 reflections with *I* > 2σ(*I*)
graphite	*R*_int_ = 0.058
Detector resolution: 13.6612 pixels mm^-1^	θ_max_ = 25.0°, θ_min_ = 1.8°
Thin–slice ω scans	*h* = −21→21
Absorption correction: multi-scan (*CrystalClear*; Rigaku, 2005)	*k* = −18→15
*T*_min_ = 0.831, *T*_max_ = 0.862	*l* = −19→20
22003 measured reflections	

### Refinement

**Table:**

Refinement on *F*^2^	Primary atom site location: structure-invariant direct methods
Least-squares matrix: full	Secondary atom site location: difference Fourier map
*R*[*F*^2^ > 2σ(*F*^2^)] = 0.038	Hydrogen site location: inferred from neighbouring sites
*wR*(*F*^2^) = 0.059	H-atom parameters constrained
*S* = 0.96	*w* = 1/[σ^2^(*F*_o_^2^) + (0.010*P*)^2^] where *P* = (*F*_o_^2^ + 2*F*_c_^2^)/3
7779 reflections	(Δ/σ)_max_ = 0.040
463 parameters	Δρ_max_ = 0.64 e Å^−^^3^
0 restraints	Δρ_min_ = −0.66 e Å^−^^3^

### Special details

**Table:**

Geometry. All esds (except the esd in the dihedral angle between two l.s. planes) are estimated using the full covariance matrix. The cell esds are taken into account individually in the estimation of esds in distances, angles and torsion angles; correlations between esds in cell parameters are only used when they are defined by crystal symmetry. An approximate (isotropic) treatment of cell esds is used for estimating esds involving l.s. planes.
Refinement. Refinement of *F*^2^ against ALL reflections. The weighted *R*-factor wR and goodness of fit *S* are based on *F*^2^, conventional *R*-factors *R* are based on *F*, with *F* set to zero for negative *F*^2^. The threshold expression of *F*^2^ > σ(*F*^2^) is used only for calculating *R*-factors(gt) etc. and is not relevant to the choice of reflections for refinement. *R*-factors based on *F*^2^ are statistically about twice as large as those based on *F*, and *R*- factors based on ALL data will be even larger.

### Fractional atomic coordinates and isotropic or equivalent isotropic displacement parameters (Å^2^)

**Table:**

	*x*	*y*	*z*	*U*_iso_*/*U*_eq_	
Br1	0.06334 (3)	0.57394 (3)	−0.16013 (4)	0.06124 (18)	
Ni2	−0.10293 (3)	0.51327 (3)	−0.23954 (4)	0.02985 (17)	
N5	−0.0695 (2)	0.4164 (2)	−0.2988 (2)	0.0339 (10)	
H5	−0.0146	0.4182	−0.2789	0.041*	
N6	−0.1135 (2)	0.5992 (2)	−0.3277 (2)	0.0352 (11)	
N7	−0.1596 (2)	0.6021 (2)	−0.1971 (2)	0.0357 (10)	
H7	−0.2127	0.5973	−0.2307	0.043*	
N8	−0.1079 (2)	0.4233 (2)	−0.1599 (2)	0.0355 (10)	
C17	−0.0961 (3)	0.4235 (3)	−0.3918 (3)	0.0394 (14)	
C18	−0.0609 (3)	0.5076 (3)	−0.4096 (3)	0.0427 (14)	
H18A	−0.0029	0.5054	−0.3792	0.051*	
H18B	−0.0700	0.5082	−0.4687	0.051*	
C19	−0.0903 (3)	0.5929 (3)	−0.3893 (3)	0.0343 (13)	
C20	−0.0899 (3)	0.6657 (3)	−0.4463 (3)	0.0555 (16)	
H20A	−0.1179	0.7142	−0.4355	0.083*	
H20B	−0.1164	0.6478	−0.5035	0.083*	
H20C	−0.0352	0.6817	−0.4364	0.083*	
C21	−0.1883 (3)	0.4229 (3)	−0.4317 (3)	0.0529 (15)	
H21A	−0.2078	0.3660	−0.4275	0.079*	
H21B	−0.2054	0.4389	−0.4896	0.079*	
H21C	−0.2096	0.4632	−0.4030	0.079*	
C22	−0.0610 (3)	0.3490 (3)	−0.4255 (3)	0.0613 (17)	
H22A	−0.0031	0.3510	−0.4007	0.092*	
H22B	−0.0783	0.3540	−0.4852	0.092*	
H22C	−0.0796	0.2952	−0.4118	0.092*	
C23	−0.1436 (3)	0.6823 (3)	−0.3104 (3)	0.0514 (15)	
H23A	−0.2003	0.6888	−0.3456	0.062*	
H23B	−0.1139	0.7292	−0.3227	0.062*	
C24	−0.1332 (3)	0.6852 (3)	−0.2194 (3)	0.0501 (15)	
H24A	−0.0769	0.6954	−0.1846	0.060*	
H24B	−0.1651	0.7317	−0.2101	0.060*	
C25	−0.1571 (3)	0.5927 (3)	−0.1092 (3)	0.0341 (13)	
C26	−0.1918 (3)	0.5040 (3)	−0.1031 (3)	0.0439 (15)	
H26A	−0.1953	0.5003	−0.0484	0.053*	
H26B	−0.2466	0.5024	−0.1442	0.053*	
C27	−0.1502 (3)	0.4240 (3)	−0.1146 (3)	0.0369 (13)	
C28	−0.1643 (3)	0.3468 (3)	−0.0697 (3)	0.0574 (16)	
H28A	−0.1586	0.2953	−0.0980	0.086*	
H28B	−0.2177	0.3493	−0.0692	0.086*	
H28C	−0.1255	0.3463	−0.0132	0.086*	
C29	−0.0720 (3)	0.6024 (3)	−0.0467 (3)	0.0507 (15)	
H29A	−0.0374	0.5626	−0.0599	0.076*	
H29B	−0.0706	0.5904	0.0085	0.076*	
H29C	−0.0537	0.6602	−0.0486	0.076*	
C30	−0.2117 (3)	0.6610 (3)	−0.0929 (3)	0.0535 (16)	
H30A	−0.1939	0.7175	−0.1011	0.080*	
H30B	−0.2089	0.6557	−0.0364	0.080*	
H30C	−0.2664	0.6525	−0.1310	0.080*	
C31	−0.0680 (3)	0.3446 (3)	−0.1725 (3)	0.0435 (14)	
H31A	−0.0861	0.2950	−0.1501	0.052*	
H31B	−0.0100	0.3496	−0.1442	0.052*	
C32	−0.0902 (3)	0.3340 (3)	−0.2661 (3)	0.0459 (14)	
H32A	−0.0602	0.2864	−0.2770	0.055*	
H32B	−0.1474	0.3220	−0.2936	0.055*	
Br4	−0.81792 (3)	0.44516 (3)	−0.34183 (4)	0.06261 (18)	
Ni1	−0.64399 (4)	0.49111 (3)	−0.26081 (4)	0.03044 (17)	
N1	−0.5901 (2)	0.3918 (2)	−0.2936 (2)	0.0354 (11)	
H1	−0.5368	0.3963	−0.2606	0.043*	
N2	−0.6377 (2)	0.5683 (2)	−0.3500 (2)	0.0368 (11)	
N3	−0.6786 (2)	0.5972 (2)	−0.2150 (2)	0.0353 (10)	
H3	−0.7336	0.5963	−0.2372	0.042*	
N4	−0.6390 (2)	0.4167 (2)	−0.1647 (2)	0.0359 (11)	
C1	−0.5930 (3)	0.3873 (3)	−0.3819 (3)	0.0313 (12)	
C2	−0.5582 (3)	0.4715 (3)	−0.3992 (3)	0.0380 (14)	
H2A	−0.5032	0.4758	−0.3589	0.046*	
H2B	−0.5551	0.4662	−0.4540	0.046*	
C3	−0.5990 (3)	0.5561 (3)	−0.3978 (3)	0.0354 (13)	
C4	−0.5878 (3)	0.6236 (3)	−0.4561 (3)	0.0611 (17)	
H4A	−0.6262	0.6139	−0.5119	0.092*	
H4B	−0.5341	0.6199	−0.4557	0.092*	
H4C	−0.5962	0.6800	−0.4377	0.092*	
C5	−0.6790 (3)	0.3733 (3)	−0.4429 (3)	0.0454 (14)	
H5A	−0.6964	0.3164	−0.4355	0.068*	
H5B	−0.6813	0.3796	−0.4991	0.068*	
H5C	−0.7138	0.4152	−0.4326	0.068*	
C6	−0.5388 (3)	0.3142 (3)	−0.3897 (3)	0.0508 (15)	
H6A	−0.4843	0.3244	−0.3517	0.076*	
H6B	−0.5406	0.3121	−0.4460	0.076*	
H6C	−0.5575	0.2604	−0.3761	0.076*	
C7	−0.6760 (3)	0.6513 (3)	−0.3461 (3)	0.0472 (14)	
H7A	−0.7341	0.6472	−0.3752	0.057*	
H7B	−0.6560	0.6962	−0.3724	0.057*	
C8	−0.6554 (3)	0.6722 (3)	−0.2541 (3)	0.0432 (14)	
H8A	−0.5980	0.6833	−0.2267	0.052*	
H8B	−0.6846	0.7231	−0.2488	0.052*	
C9	−0.6561 (3)	0.6020 (3)	−0.1223 (3)	0.0371 (13)	
C10	−0.6925 (3)	0.5229 (3)	−0.0965 (3)	0.0429 (15)	
H10A	−0.6861	0.5306	−0.0385	0.051*	
H10B	−0.7501	0.5230	−0.1295	0.051*	
C11	−0.6607 (3)	0.4350 (3)	−0.1044 (3)	0.0351 (13)	
C12	−0.6587 (3)	0.3714 (3)	−0.0382 (3)	0.0623 (17)	
H12A	−0.7127	0.3529	−0.0476	0.094*	
H12B	−0.6354	0.3981	0.0159	0.094*	
H12C	−0.6267	0.3225	−0.0405	0.094*	
C13	−0.5648 (3)	0.6037 (3)	−0.0775 (3)	0.0556 (15)	
H13A	−0.5413	0.5583	−0.0988	0.083*	
H13B	−0.5511	0.5955	−0.0186	0.083*	
H13C	−0.5443	0.6583	−0.0869	0.083*	
C14	−0.6937 (3)	0.6820 (3)	−0.0992 (3)	0.0593 (17)	
H14A	−0.6707	0.7330	−0.1129	0.089*	
H14B	−0.6829	0.6814	−0.0403	0.089*	
H14C	−0.7509	0.6818	−0.1300	0.089*	
C15	−0.6085 (3)	0.3292 (3)	−0.1724 (3)	0.0501 (15)	
H15A	−0.6381	0.2860	−0.1548	0.060*	
H15B	−0.5518	0.3247	−0.1367	0.060*	
C16	−0.6193 (3)	0.3139 (3)	−0.2628 (3)	0.0491 (16)	
H16A	−0.5887	0.2636	−0.2669	0.059*	
H16B	−0.6758	0.3038	−0.2965	0.059*	
O1	−0.1258 (2)	0.14016 (19)	−0.1866 (2)	0.0825 (13)	
H1C	−0.1085	0.1305	−0.2255	0.124*	
H1D	−0.1424	0.0927	−0.1741	0.124*	
O2	−0.5584 (2)	0.0948 (2)	−0.2991 (2)	0.0977 (15)	
H2E	−0.5670	0.0435	−0.3176	0.147*	
H2F	−0.5789	0.1006	−0.2622	0.147*	
O3	−0.3103 (2)	0.3868 (2)	−0.2944 (2)	0.0893 (13)	
H3C	−0.3183	0.4385	−0.3121	0.134*	
H3D	−0.3299	0.3811	−0.2568	0.134*	
O4	−0.1221 (2)	0.8839 (2)	−0.3111 (3)	0.1020 (16)	
H4F	−0.1049	0.9311	−0.3232	0.153*	
H4G	−0.1388	0.8935	−0.2718	0.153*	
Br2	−0.64785 (4)	0.09231 (3)	−0.15400 (4)	0.06252 (18)	
Br3	−0.39677 (4)	0.38931 (4)	−0.14455 (4)	0.06808 (19)	

### Atomic displacement parameters (Å^2^)

**Table:**

	*U*^11^	*U*^22^	*U*^33^	*U*^12^	*U*^13^	*U*^23^
Br1	0.0463 (4)	0.0723 (4)	0.0632 (5)	−0.0046 (3)	0.0184 (4)	−0.0208 (3)
Ni2	0.0436 (5)	0.0205 (3)	0.0252 (4)	0.0034 (3)	0.0127 (4)	0.0004 (3)
N5	0.032 (3)	0.034 (2)	0.031 (3)	−0.0005 (18)	0.006 (2)	−0.0054 (19)
N6	0.045 (3)	0.025 (2)	0.035 (3)	0.0054 (18)	0.015 (2)	−0.001 (2)
N7	0.040 (3)	0.031 (2)	0.035 (3)	−0.0004 (18)	0.013 (2)	−0.003 (2)
N8	0.041 (3)	0.029 (2)	0.033 (3)	0.0009 (19)	0.011 (2)	−0.0011 (19)
C17	0.050 (4)	0.041 (3)	0.021 (3)	0.000 (3)	0.006 (3)	−0.003 (3)
C18	0.044 (4)	0.048 (3)	0.035 (3)	−0.003 (3)	0.013 (3)	−0.003 (3)
C19	0.032 (3)	0.031 (3)	0.032 (3)	−0.008 (2)	0.002 (3)	−0.003 (3)
C20	0.075 (5)	0.047 (3)	0.045 (4)	−0.006 (3)	0.023 (4)	0.007 (3)
C21	0.049 (4)	0.054 (3)	0.040 (4)	−0.009 (3)	−0.001 (3)	−0.004 (3)
C22	0.087 (5)	0.050 (3)	0.051 (4)	0.003 (3)	0.031 (4)	−0.016 (3)
C23	0.076 (4)	0.032 (3)	0.048 (4)	0.009 (3)	0.026 (4)	0.006 (3)
C24	0.072 (4)	0.034 (3)	0.052 (4)	0.002 (3)	0.032 (4)	−0.003 (3)
C25	0.034 (4)	0.033 (3)	0.036 (4)	−0.002 (2)	0.013 (3)	−0.006 (2)
C26	0.043 (4)	0.047 (3)	0.046 (4)	−0.005 (3)	0.022 (3)	−0.003 (3)
C27	0.040 (4)	0.033 (3)	0.029 (3)	−0.007 (2)	0.004 (3)	0.001 (2)
C28	0.074 (5)	0.048 (3)	0.054 (4)	−0.009 (3)	0.028 (4)	0.007 (3)
C29	0.046 (4)	0.059 (3)	0.039 (4)	−0.004 (3)	0.006 (3)	−0.007 (3)
C30	0.052 (4)	0.052 (3)	0.057 (4)	0.002 (3)	0.020 (3)	−0.014 (3)
C31	0.053 (4)	0.032 (3)	0.044 (4)	0.010 (2)	0.018 (3)	0.012 (3)
C32	0.063 (4)	0.030 (3)	0.047 (4)	0.003 (3)	0.023 (3)	−0.001 (3)
Br4	0.0479 (4)	0.0731 (4)	0.0598 (4)	−0.0013 (3)	0.0123 (3)	−0.0233 (3)
Ni1	0.0474 (5)	0.0200 (3)	0.0248 (4)	0.0021 (3)	0.0146 (4)	0.0008 (3)
N1	0.046 (3)	0.029 (2)	0.032 (3)	0.0045 (19)	0.016 (2)	0.0023 (19)
N2	0.049 (3)	0.025 (2)	0.034 (3)	0.0060 (19)	0.013 (2)	0.0005 (19)
N3	0.042 (3)	0.033 (2)	0.025 (3)	0.0006 (19)	0.007 (2)	−0.003 (2)
N4	0.050 (3)	0.025 (2)	0.034 (3)	0.0023 (19)	0.017 (2)	0.0007 (19)
C1	0.032 (4)	0.034 (3)	0.027 (3)	−0.003 (2)	0.011 (3)	−0.005 (2)
C2	0.038 (4)	0.044 (3)	0.035 (3)	0.002 (3)	0.017 (3)	−0.004 (3)
C3	0.036 (4)	0.040 (3)	0.026 (3)	−0.009 (3)	0.006 (3)	0.002 (3)
C4	0.067 (5)	0.064 (4)	0.061 (4)	0.011 (3)	0.035 (4)	0.027 (3)
C5	0.049 (4)	0.045 (3)	0.041 (4)	−0.001 (3)	0.015 (3)	−0.005 (3)
C6	0.057 (4)	0.044 (3)	0.052 (4)	0.012 (3)	0.020 (3)	−0.010 (3)
C7	0.069 (4)	0.032 (3)	0.045 (4)	0.005 (3)	0.027 (3)	0.006 (3)
C8	0.070 (4)	0.022 (3)	0.038 (4)	0.001 (2)	0.021 (3)	−0.001 (3)
C9	0.049 (4)	0.034 (3)	0.027 (3)	−0.004 (3)	0.014 (3)	−0.005 (2)
C10	0.043 (4)	0.048 (3)	0.041 (4)	−0.005 (3)	0.020 (3)	−0.004 (3)
C11	0.032 (3)	0.040 (3)	0.028 (3)	−0.007 (2)	0.006 (3)	−0.001 (3)
C12	0.086 (5)	0.060 (4)	0.052 (4)	0.006 (3)	0.039 (4)	0.016 (3)
C13	0.052 (4)	0.063 (4)	0.037 (4)	−0.024 (3)	0.000 (3)	−0.003 (3)
C14	0.083 (5)	0.050 (4)	0.048 (4)	0.001 (3)	0.028 (4)	−0.013 (3)
C15	0.077 (4)	0.036 (3)	0.043 (4)	−0.001 (3)	0.029 (3)	0.011 (3)
C16	0.075 (5)	0.024 (3)	0.056 (4)	−0.003 (3)	0.034 (4)	0.000 (3)
O1	0.089 (3)	0.058 (2)	0.102 (3)	−0.002 (2)	0.038 (3)	−0.006 (2)
O2	0.105 (4)	0.098 (3)	0.099 (4)	−0.012 (3)	0.049 (3)	0.033 (3)
O3	0.101 (4)	0.070 (3)	0.096 (4)	0.008 (2)	0.036 (3)	−0.022 (2)
O4	0.113 (4)	0.050 (2)	0.172 (5)	0.001 (2)	0.087 (4)	−0.003 (3)
Br2	0.0602 (4)	0.0637 (4)	0.0547 (4)	0.0070 (3)	0.0113 (4)	−0.0054 (3)
Br3	0.0607 (5)	0.0799 (4)	0.0528 (4)	−0.0097 (3)	0.0090 (4)	0.0079 (3)

### Geometric parameters (Å, °)

**Table:**

Ni2—N6	1.976 (4)	N1—C16	1.490 (4)
Ni2—N8	1.983 (3)	N1—C1	1.504 (5)
Ni2—N7	2.003 (3)	N1—H1	0.9101
Ni2—N5	2.029 (3)	N2—C3	1.272 (5)
N5—C17	1.495 (5)	N2—C7	1.471 (5)
N5—C32	1.497 (5)	N3—C8	1.479 (4)
N5—H5	0.9100	N3—C9	1.495 (5)
N6—C19	1.280 (5)	N3—H3	0.9099
N6—C23	1.469 (5)	N4—C11	1.271 (5)
N7—C24	1.472 (5)	N4—C15	1.486 (5)
N7—C25	1.505 (5)	C1—C5	1.516 (6)
N7—H7	0.9100	C1—C2	1.524 (5)
N8—C27	1.275 (5)	C1—C6	1.528 (5)
N8—C31	1.470 (5)	C2—C3	1.504 (5)
C17—C21	1.529 (6)	C2—H2A	0.9700
C17—C18	1.527 (6)	C2—H2B	0.9700
C17—C22	1.529 (5)	C3—C4	1.516 (5)
C18—C19	1.512 (6)	C4—H4A	0.9600
C18—H18A	0.9700	C4—H4B	0.9600
C18—H18B	0.9700	C4—H4C	0.9600
C19—C20	1.497 (5)	C5—H5A	0.9600
C20—H20A	0.9600	C5—H5B	0.9600
C20—H20B	0.9600	C5—H5C	0.9600
C20—H20C	0.9600	C6—H6A	0.9600
C21—H21A	0.9600	C6—H6B	0.9600
C21—H21B	0.9600	C6—H6C	0.9600
C21—H21C	0.9600	C7—C8	1.521 (5)
C22—H22A	0.9600	C7—H7A	0.9700
C22—H22B	0.9600	C7—H7B	0.9700
C22—H22C	0.9600	C8—H8A	0.9700
C23—C24	1.509 (6)	C8—H8B	0.9700
C23—H23A	0.9700	C9—C13	1.520 (6)
C23—H23B	0.9700	C9—C10	1.531 (5)
C24—H24A	0.9700	C9—C14	1.533 (5)
C24—H24B	0.9700	C10—C11	1.502 (6)
C25—C29	1.505 (6)	C10—H10A	0.9700
C25—C26	1.529 (5)	C10—H10B	0.9700
C25—C30	1.536 (5)	C11—C12	1.497 (5)
C26—C27	1.496 (6)	C12—H12A	0.9600
C26—H26A	0.9700	C12—H12B	0.9600
C26—H26B	0.9700	C12—H12C	0.9600
C27—C28	1.496 (5)	C13—H13A	0.9600
C28—H28A	0.9600	C13—H13B	0.9600
C28—H28B	0.9600	C13—H13C	0.9600
C28—H28C	0.9600	C14—H14A	0.9600
C29—H29A	0.9600	C14—H14B	0.9600
C29—H29B	0.9600	C14—H14C	0.9600
C29—H29C	0.9600	C15—C16	1.515 (5)
C30—H30A	0.9600	C15—H15A	0.9700
C30—H30B	0.9600	C15—H15B	0.9700
C30—H30C	0.9600	C16—H16A	0.9700
C31—C32	1.518 (5)	C16—H16B	0.9700
C31—H31A	0.9700	O1—H1C	0.8500
C31—H31B	0.9700	O1—H1D	0.8500
C32—H32A	0.9700	O2—H2E	0.8499
C32—H32B	0.9700	O2—H2F	0.8500
Ni1—N2	1.985 (4)	O3—H3C	0.8498
Ni1—N4	1.993 (4)	O3—H3D	0.8500
Ni1—N1	2.007 (3)	O4—H4F	0.8501
Ni1—N3	2.020 (3)	O4—H4G	0.8499
			
N6—Ni2—N8	172.20 (16)	N4—Ni1—N1	85.03 (14)
N6—Ni2—N7	84.38 (14)	N2—Ni1—N3	85.80 (14)
N8—Ni2—N7	94.04 (14)	N4—Ni1—N3	94.09 (14)
N6—Ni2—N5	94.20 (14)	N1—Ni1—N3	170.06 (17)
N8—Ni2—N5	85.74 (14)	C16—N1—C1	116.0 (3)
N7—Ni2—N5	167.87 (17)	C16—N1—Ni1	104.6 (2)
C17—N5—C32	116.3 (3)	C1—N1—Ni1	118.9 (3)
C17—N5—Ni2	116.7 (3)	C16—N1—H1	105.3
C32—N5—Ni2	106.3 (2)	C1—N1—H1	105.4
C17—N5—H5	105.4	Ni1—N1—H1	105.4
C32—N5—H5	105.6	C3—N2—C7	122.5 (4)
Ni2—N5—H5	105.5	C3—N2—Ni1	127.9 (3)
C19—N6—C23	119.6 (4)	C7—N2—Ni1	109.1 (3)
C19—N6—Ni2	128.2 (3)	C8—N3—C9	116.0 (3)
C23—N6—Ni2	111.8 (3)	C8—N3—Ni1	106.4 (2)
C24—N7—C25	117.1 (3)	C9—N3—Ni1	117.3 (3)
C24—N7—Ni2	104.5 (3)	C8—N3—H3	105.3
C25—N7—Ni2	118.2 (3)	C9—N3—H3	105.3
C24—N7—H7	105.4	Ni1—N3—H3	105.3
C25—N7—H7	105.2	C11—N4—C15	120.8 (4)
Ni2—N7—H7	105.2	C11—N4—Ni1	128.2 (3)
C27—N8—C31	122.0 (4)	C15—N4—Ni1	110.9 (3)
C27—N8—Ni2	127.5 (3)	N1—C1—C5	110.4 (4)
C31—N8—Ni2	109.3 (3)	N1—C1—C2	107.3 (3)
N5—C17—C21	109.6 (4)	C5—C1—C2	111.6 (4)
N5—C17—C18	106.8 (4)	N1—C1—C6	109.9 (4)
C21—C17—C18	111.8 (4)	C5—C1—C6	110.1 (4)
N5—C17—C22	109.9 (4)	C2—C1—C6	107.4 (4)
C21—C17—C22	110.5 (4)	C3—C2—C1	120.5 (4)
C18—C17—C22	108.1 (4)	C3—C2—H2A	107.2
C19—C18—C17	119.7 (4)	C1—C2—H2A	107.2
C19—C18—H18A	107.4	C3—C2—H2B	107.2
C17—C18—H18A	107.4	C1—C2—H2B	107.2
C19—C18—H18B	107.4	H2A—C2—H2B	106.8
C17—C18—H18B	107.4	N2—C3—C2	121.7 (4)
H18A—C18—H18B	106.9	N2—C3—C4	124.4 (4)
N6—C19—C20	124.6 (4)	C2—C3—C4	114.0 (4)
N6—C19—C18	120.5 (4)	C3—C4—H4A	109.5
C20—C19—C18	114.8 (4)	C3—C4—H4B	109.5
C19—C20—H20A	109.5	H4A—C4—H4B	109.5
C19—C20—H20B	109.5	C3—C4—H4C	109.5
H20A—C20—H20B	109.5	H4A—C4—H4C	109.5
C19—C20—H20C	109.5	H4B—C4—H4C	109.5
H20A—C20—H20C	109.5	C1—C5—H5A	109.5
H20B—C20—H20C	109.5	C1—C5—H5B	109.5
C17—C21—H21A	109.5	H5A—C5—H5B	109.5
C17—C21—H21B	109.5	C1—C5—H5C	109.5
H21A—C21—H21B	109.5	H5A—C5—H5C	109.5
C17—C21—H21C	109.5	H5B—C5—H5C	109.5
H21A—C21—H21C	109.5	C1—C6—H6A	109.5
H21B—C21—H21C	109.5	C1—C6—H6B	109.5
C17—C22—H22A	109.5	H6A—C6—H6B	109.5
C17—C22—H22B	109.5	C1—C6—H6C	109.5
H22A—C22—H22B	109.5	H6A—C6—H6C	109.5
C17—C22—H22C	109.5	H6B—C6—H6C	109.5
H22A—C22—H22C	109.5	N2—C7—C8	107.4 (4)
H22B—C22—H22C	109.5	N2—C7—H7A	110.2
N6—C23—C24	109.0 (4)	C8—C7—H7A	110.2
N6—C23—H23A	109.9	N2—C7—H7B	110.2
C24—C23—H23A	109.9	C8—C7—H7B	110.2
N6—C23—H23B	109.9	H7A—C7—H7B	108.5
C24—C23—H23B	109.9	N3—C8—C7	107.9 (3)
H23A—C23—H23B	108.3	N3—C8—H8A	110.1
N7—C24—C23	108.6 (4)	C7—C8—H8A	110.1
N7—C24—H24A	110.0	N3—C8—H8B	110.1
C23—C24—H24A	110.0	C7—C8—H8B	110.1
N7—C24—H24B	110.0	H8A—C8—H8B	108.4
C23—C24—H24B	110.0	N3—C9—C13	110.5 (4)
H24A—C24—H24B	108.4	N3—C9—C10	107.3 (4)
N7—C25—C29	110.6 (4)	C13—C9—C10	111.1 (4)
N7—C25—C26	107.3 (4)	N3—C9—C14	110.4 (4)
C29—C25—C26	111.5 (4)	C13—C9—C14	110.2 (4)
N7—C25—C30	109.7 (4)	C10—C9—C14	107.3 (4)
C29—C25—C30	109.9 (4)	C11—C10—C9	118.9 (4)
C26—C25—C30	107.8 (4)	C11—C10—H10A	107.6
C27—C26—C25	120.2 (4)	C9—C10—H10A	107.6
C27—C26—H26A	107.3	C11—C10—H10B	107.6
C25—C26—H26A	107.3	C9—C10—H10B	107.6
C27—C26—H26B	107.3	H10A—C10—H10B	107.0
C25—C26—H26B	107.3	N4—C11—C12	123.3 (4)
H26A—C26—H26B	106.9	N4—C11—C10	121.3 (4)
N8—C27—C28	124.2 (4)	C12—C11—C10	115.5 (4)
N8—C27—C26	121.8 (4)	C11—C12—H12A	109.5
C28—C27—C26	114.0 (4)	C11—C12—H12B	109.5
C27—C28—H28A	109.5	H12A—C12—H12B	109.5
C27—C28—H28B	109.5	C11—C12—H12C	109.5
H28A—C28—H28B	109.5	H12A—C12—H12C	109.5
C27—C28—H28C	109.5	H12B—C12—H12C	109.5
H28A—C28—H28C	109.5	C9—C13—H13A	109.5
H28B—C28—H28C	109.5	C9—C13—H13B	109.5
C25—C29—H29A	109.5	H13A—C13—H13B	109.5
C25—C29—H29B	109.5	C9—C13—H13C	109.5
H29A—C29—H29B	109.5	H13A—C13—H13C	109.5
C25—C29—H29C	109.5	H13B—C13—H13C	109.5
H29A—C29—H29C	109.5	C9—C14—H14A	109.5
H29B—C29—H29C	109.5	C9—C14—H14B	109.5
C25—C30—H30A	109.5	H14A—C14—H14B	109.5
C25—C30—H30B	109.5	C9—C14—H14C	109.5
H30A—C30—H30B	109.5	H14A—C14—H14C	109.5
C25—C30—H30C	109.5	H14B—C14—H14C	109.5
H30A—C30—H30C	109.5	N4—C15—C16	109.0 (4)
H30B—C30—H30C	109.5	N4—C15—H15A	109.9
N8—C31—C32	107.4 (4)	C16—C15—H15A	109.9
N8—C31—H31A	110.2	N4—C15—H15B	109.9
C32—C31—H31A	110.2	C16—C15—H15B	109.9
N8—C31—H31B	110.2	H15A—C15—H15B	108.3
C32—C31—H31B	110.2	N1—C16—C15	108.2 (4)
H31A—C31—H31B	108.5	N1—C16—H16A	110.1
N5—C32—C31	107.7 (3)	C15—C16—H16A	110.1
N5—C32—H32A	110.2	N1—C16—H16B	110.1
C31—C32—H32A	110.2	C15—C16—H16B	110.1
N5—C32—H32B	110.2	H16A—C16—H16B	108.4
C31—C32—H32B	110.2	H1C—O1—H1D	108.1
H32A—C32—H32B	108.5	H2E—O2—H2F	108.1
N2—Ni1—N4	174.32 (16)	H3C—O3—H3D	108.1
N2—Ni1—N1	94.09 (14)	H4F—O4—H4G	108.1
			
N6—Ni2—N5—C17	−25.0 (3)	N2—Ni1—N1—C16	−155.2 (3)
N8—Ni2—N5—C17	147.2 (3)	N4—Ni1—N1—C16	30.5 (3)
N7—Ni2—N5—C17	57.8 (8)	N3—Ni1—N1—C16	115.8 (8)
N6—Ni2—N5—C32	−156.6 (3)	N2—Ni1—N1—C1	−23.8 (3)
N8—Ni2—N5—C32	15.6 (3)	N4—Ni1—N1—C1	161.8 (3)
N7—Ni2—N5—C32	−73.8 (8)	N3—Ni1—N1—C1	−112.8 (9)
N8—Ni2—N6—C19	−100.7 (12)	N4—Ni1—N2—C3	71.0 (17)
N7—Ni2—N6—C19	−179.4 (4)	N1—Ni1—N2—C3	−9.9 (4)
N5—Ni2—N6—C19	−11.5 (4)	N3—Ni1—N2—C3	160.1 (4)
N8—Ni2—N6—C23	86.9 (12)	N4—Ni1—N2—C7	−100.7 (16)
N7—Ni2—N6—C23	8.3 (3)	N1—Ni1—N2—C7	178.3 (3)
N5—Ni2—N6—C23	176.2 (3)	N3—Ni1—N2—C7	−11.7 (3)
N6—Ni2—N7—C24	−31.8 (3)	N2—Ni1—N3—C8	−17.3 (3)
N8—Ni2—N7—C24	155.8 (3)	N4—Ni1—N3—C8	157.0 (3)
N5—Ni2—N7—C24	−115.6 (7)	N1—Ni1—N3—C8	72.4 (10)
N6—Ni2—N7—C25	−164.0 (3)	N2—Ni1—N3—C9	−149.1 (3)
N8—Ni2—N7—C25	23.6 (3)	N4—Ni1—N3—C9	25.2 (3)
N5—Ni2—N7—C25	112.2 (7)	N1—Ni1—N3—C9	−59.3 (10)
N6—Ni2—N8—C27	−64.9 (13)	N2—Ni1—N4—C11	95.4 (17)
N7—Ni2—N8—C27	13.1 (4)	N1—Ni1—N4—C11	176.8 (4)
N5—Ni2—N8—C27	−154.7 (4)	N3—Ni1—N4—C11	6.7 (4)
N6—Ni2—N8—C31	103.2 (12)	N2—Ni1—N4—C15	−87.3 (16)
N7—Ni2—N8—C31	−178.8 (3)	N1—Ni1—N4—C15	−5.9 (3)
N5—Ni2—N8—C31	13.4 (3)	N3—Ni1—N4—C15	−176.0 (3)
C32—N5—C17—C21	64.6 (5)	C16—N1—C1—C5	60.0 (5)
Ni2—N5—C17—C21	−62.3 (4)	Ni1—N1—C1—C5	−66.1 (4)
C32—N5—C17—C18	−174.1 (3)	C16—N1—C1—C2	−178.2 (4)
Ni2—N5—C17—C18	59.0 (4)	Ni1—N1—C1—C2	55.7 (4)
C32—N5—C17—C22	−57.1 (5)	C16—N1—C1—C6	−61.7 (5)
Ni2—N5—C17—C22	176.1 (3)	Ni1—N1—C1—C6	172.2 (3)
N5—C17—C18—C19	−66.8 (5)	N1—C1—C2—C3	−62.9 (5)
C21—C17—C18—C19	53.1 (6)	C5—C1—C2—C3	58.2 (5)
C22—C17—C18—C19	175.0 (4)	C6—C1—C2—C3	179.0 (4)
C23—N6—C19—C20	1.0 (7)	C7—N2—C3—C2	178.3 (4)
Ni2—N6—C19—C20	−170.8 (3)	Ni1—N2—C3—C2	7.6 (7)
C23—N6—C19—C18	−178.5 (4)	C7—N2—C3—C4	−0.3 (7)
Ni2—N6—C19—C18	9.6 (7)	Ni1—N2—C3—C4	−171.1 (3)
C17—C18—C19—N6	31.5 (7)	C1—C2—C3—N2	31.3 (7)
C17—C18—C19—C20	−148.2 (4)	C1—C2—C3—C4	−149.9 (4)
C19—N6—C23—C24	−155.9 (4)	C3—N2—C7—C8	−134.8 (5)
Ni2—N6—C23—C24	17.2 (5)	Ni1—N2—C7—C8	37.5 (4)
C25—N7—C24—C23	−177.7 (4)	C9—N3—C8—C7	174.6 (4)
Ni2—N7—C24—C23	49.4 (4)	Ni1—N3—C8—C7	42.1 (4)
N6—C23—C24—N7	−44.5 (5)	N2—C7—C8—N3	−53.3 (5)
C24—N7—C25—C29	−61.5 (5)	C8—N3—C9—C13	−64.1 (5)
Ni2—N7—C25—C29	64.9 (4)	Ni1—N3—C9—C13	63.1 (4)
C24—N7—C25—C26	176.7 (4)	C8—N3—C9—C10	174.7 (4)
Ni2—N7—C25—C26	−56.9 (4)	Ni1—N3—C9—C10	−58.1 (5)
C24—N7—C25—C30	59.9 (5)	C8—N3—C9—C14	58.1 (5)
Ni2—N7—C25—C30	−173.7 (3)	Ni1—N3—C9—C14	−174.7 (3)
N7—C25—C26—C27	63.1 (5)	N3—C9—C10—C11	67.1 (5)
C29—C25—C26—C27	−58.1 (6)	C13—C9—C10—C11	−53.8 (6)
C30—C25—C26—C27	−178.9 (4)	C14—C9—C10—C11	−174.3 (4)
C31—N8—C27—C28	−0.1 (7)	C15—N4—C11—C12	−1.0 (7)
Ni2—N8—C27—C28	166.6 (3)	Ni1—N4—C11—C12	176.1 (3)
C31—N8—C27—C26	−179.0 (4)	C15—N4—C11—C10	−179.3 (4)
Ni2—N8—C27—C26	−12.3 (7)	Ni1—N4—C11—C10	−2.2 (7)
C25—C26—C27—N8	−28.2 (7)	C9—C10—C11—N4	−36.3 (7)
C25—C26—C27—C28	152.8 (4)	C9—C10—C11—C12	145.3 (5)
C27—N8—C31—C32	129.6 (5)	C11—N4—C15—C16	157.4 (4)
Ni2—N8—C31—C32	−39.3 (4)	Ni1—N4—C15—C16	−20.2 (5)
C17—N5—C32—C31	−172.7 (4)	C1—N1—C16—C15	177.7 (4)
Ni2—N5—C32—C31	−40.8 (4)	Ni1—N1—C16—C15	−49.3 (4)
N8—C31—C32—N5	53.4 (5)	N4—C15—C16—N1	46.3 (5)

### Hydrogen-bond geometry (Å, °)

**Table:**

*D*—H···*A*	*D*—H	H···*A*	*D*···*A*	*D*—H···*A*
N1—H1···Br3	0.91	2.55	3.444 (4)	167
O2—H2F···Br2	0.85	2.60	3.437 (3)	171
O3—H3D···Br3	0.85	2.63	3.473 (3)	171
N5—H5···O4^i^	0.91	2.41	3.269 (5)	158
O1—H1C···Br1^i^	0.85	2.55	3.390 (3)	170
N7—H7···Br2^ii^	0.91	2.54	3.436 (4)	169
N3—H3···O1^ii^	0.91	2.45	3.328 (5)	161
O2—H2E···Br3^iii^	0.85	2.50	3.339 (4)	171
O1—H1D···Br4^iii^	0.85	2.44	3.280 (3)	169
O3—H3C···Br2^ii^	0.85	2.48	3.316 (3)	170
O4—H4G···Br4^ii^	0.85	2.49	3.335 (4)	171
O4—H4F···Br1^iv^	0.85	2.39	3.228 (3)	171

Symmetry codes: (i) −*x*, *y*−1/2, −*z*−1/2; (ii) −*x*−1, *y*+1/2, −*z*−1/2; (iii) −*x*−1, *y*−1/2, −*z*−1/2; (iv) −*x*, *y*+1/2, −*z*−1/2.
